# BRCA2, EGFR, and NTRK mutations in mismatch repair-deficient colorectal cancers with MSH2 or MLH1 mutations

**DOI:** 10.18632/oncotarget.18098

**Published:** 2017-05-23

**Authors:** Safoora Deihimi, Avital Lev, Michael Slifker, Elena Shagisultanova, Qifang Xu, Kyungsuk Jung, Namrata Vijayvergia, Eric A. Ross, Joanne Xiu, Jeffrey Swensen, Zoran Gatalica, Mark Andrake, Roland L. Dunbrack, Wafik S. El-Deiry

**Affiliations:** ^1^ Laboratory of Translational Oncology and Experimental Cancer Therapeutics, Fox Chase Cancer Center, Philadelphia, PA, USA; ^2^ Molecular Therapeutics Program, Fox Chase Cancer Center, Philadelphia, PA, USA; ^3^ Department of Hematology/Oncology, Fox Chase Cancer Center, Philadelphia, PA, USA; ^4^ Biostatistics and Bioinformatics Department, Fox Chase Cancer Center, Philadelphia, PA, USA; ^5^ Department of Medicine, Fox Chase Cancer Center, Philadelphia, PA, USA; ^6^ Cancer Prevention and Control Program, Fox Chase Cancer Center, Philadelphia, PA, USA; ^7^ Department of Biochemistry and Molecular Biology, Drexel University College of Medicine, Philadelphia, PA, USA; ^8^ Caris Life Science, Phoenix, AZ, USA; ^9^ University of Colorado Denver Cancer Center, Denver, CO, USA

**Keywords:** BRCA2, EGFR, NTRK, colorectal cancer, MLH1

## Abstract

Deficient mismatch repair (MMR) and microsatellite instability (MSI) contribute to ~15% of colorectal cancer (CRCs). We hypothesized MSI leads to mutations in DNA repair proteins including BRCA2 and cancer drivers including EGFR. We analyzed mutations among a discovery cohort of 26 MSI-High (MSI-H) and 558 non-MSI-H CRCs profiled at Caris Life Sciences. Caris-profiled MSI-H CRCs had high mutation rates (50% vs 14% in non-MSI-H, *P* < 0.0001) in BRCA2. Of 1104 profiled CRCs from a second cohort (COSMIC), MSH2/MLH1-mutant CRCs showed higher mutation rates in BRCA2 compared to non-MSH2/MLH1-mutant tumors (38% vs 6%, *P* < 0.0000001). BRCA2 mutations in MSH2/MLH1-mutant CRCs included 75 unique mutations not known to occur in breast or pancreatic cancer per COSMIC v73. Only 5 deleterious BRCA2 mutations in CRC were previously reported in the BIC database as germ-line mutations in breast cancer. Some BRCA2 mutations were predicted to disrupt interactions with partner proteins DSS1 and RAD51. Some CRCs harbored multiple BRCA2 mutations. EGFR was mutated in 45.5% of MSH2/MLH1-mutant and 6.5% of non-MSH2/MLH1-mutant tumors (*P* < 0.0000001). Approximately 15% of EGFR mutations found may be actionable through TKI therapy, including N700D, G719D, T725M, T790M, and E884K. NTRK gene mutations were identified in MSH2/MLH1-mutant CRC including NTRK1 I699V, NTRK2 P716S, and NTRK3 R745L. Our findings have clinical relevance regarding therapeutic targeting of BRCA2 vulnerabilities, EGFR mutations or other identified oncogenic drivers such as NTRK in MSH2/MLH1-mutant CRCs or other tumors with mismatch repair deficiency.

## INTRODUCTION

Colorectal cancer (CRC) incidence and mortality rates are remarkably high worldwide, with 1.4 million new cases and approximately 700,000 deaths per year [1]. With the rapid increase, the global incidence and mortality rate of CRC is predicted to undergo a 60% rise by 2030 [2]. CRCs arise through genetic changes that impact various driver genes or increased mutation rates in microsatellite unstable tumors [3, 4]. A hypermutable phenotype associated with microsatellite instability (MSI) results from loss of mismatch repair (MMR) activity [5, 6]. MSI is detected in a small fraction (<15%) of all CRCs, and such tumors have a better prognosis and different chemotherapeutic sensitivities as compared to non-MSI tumors [3, 5, 7, 8]. As such, MSI is less frequently found in advanced CRCs where they occur with a frequency of ~4% [9].

Approximately 90% of hereditary non-polyposis colorectal cancer (HPNCC) patients are reported to harbor germ-line mutations in MLH1 and MSH2 [3, 10]. Germ-line, somatic and epigenetic inactivation of the MMR genes MLH1 and MSH2 results in complete loss of MMR leading to oncogenesis, recognized as an MSI-H state both sporadically and in HPNCC [3, 11]. A distinct MSI phenotype with a low level of the MMR markers MSH3, MSH6, PMS1 and PMS2 is known as the MSI-Low (MSI-L) CRC subtype with a weak effect towards MMR system failure [3, 5, 6]. The MMR pathway functions as an essential system for maintenance of genome integrity, and also mediates DNA double-strand break (DSB) repair [11]. Various studies have suggested a modulator effect of MSH2 and MLH1 in homologous recombination (HR) [11, 12]. Delays in the recruitment of RAD51 and MRE11 to DNA damage sites, and failed repair of DNA DSBs mediated by gene conversion is observed in MSH2-deficient colorectal cancer cells [11, 12]. Ionizing radiation can induce a high frequency of mitotic recombination in MLH1-null cells [11, 13]. How mechanistically MSH2 and MLH1 impact on DSB repair and HR factors remains to be fully understood [14].

Repetitive DNA sequences are more prone to mutation in tumors with MMR deficiency [7]. Coding microsatellites in HR factors hRAD50 and MRE11A can be mutated in MSI tumors and are reported to sensitize MSI tumors to PARP-1 inhibitors [7]. Repetitive sequences within the Bax or TGF-beta Type II receptor genes have been reported to be mutated in MMR-deficient CRCs [15]. The BRCA2 protein is a fundamental element of HR and somatic mutations in BRCA2 are known cancer drivers [16, 17]. There is little evidence to suggest that BRCA2 mutations are associated with increased risk of colon cancer although it is known that BRCA1 mutation carriers have about a 3-fold increased risk of CRC [18, 19]. A recent study has implicated an association between BRCA2 mutations and risk of CRC [20]. The high frequency of repetitive sequences in BRCA2 could allow for frequent mutations in MSI tumors [21]. Identification of somatic mutations in BRCA2 could provide a basis for therapy with PARP-1 inhibitors especially if the defects are biallelic [22]. We hypothesized that BRCA2, because of its high frequency of microsatellites, may be a substrate for mutation and may lead to a driver phenotype in tumors that have lost their MMR system and there is potential to acquire biallelic hits in BRCA2.

Epidermal growth factor receptor (EGFR), a member of the ErbB family, is a transmembrane glycoprotein that forms a receptor tyrosine kinase [23, 24]. EGFR overexpression is associated with tumorigenesis and malignancy of many epithelial tumors including MSI colon cancer through activation of downstream signaling pathways involving RAS-RAF-MAPK and PTEN-PI3K-AKT [24, 25]. EGFR mutations in the tyrosine kinase domain occur in ~10% of Non-small cell lung cancer (NSCLC), and sensitize the patients’ tumors to tyrosine kinase inhibitors (TKI) [26]. These patients are typically non-smokers, female and of Asian descent. There are limited reports on the incidence rate of EGFR mutation in colorectal patients [24, 27, 28]. Here, we further analyzed the mutation signature of EGFR for possible targeted therapy.

NTRK fusions have been identified and can act as drivers rarely in common tumors (CRC where they were originally discovered, breast, lung cancer, GBM, and others) or more frequently in some less common tumors (papillary thyroid cancers, congenital mesoblastic nephroma, pontine glioma, secretory breast carcinoma, and mammary analogue secretory carcinoma (MASC) of the salivary glands) [29–32]. In addition to fusions, NTRK gene mutations have been described [33]. Small molecule inhibitors of NTRK kinase activity are being tested in multiple clinical trials [34–37].

Advanced MSI-H CRCs have recently been found to respond to immune checkpoint therapy although not all patients respond and most do not have durable responses at least with single agent therapy [38]. Our results begin to identify additional vulnerabilities in MSI-H or MSH2/MLH1-mutant CRCs that may be considered in designing therapeutic options for patients with advanced CRC, and the insights may apply to other tumors with mismatch repair deficiency.

## RESULTS

### Discovery cohort of CRC analyzed by genomic profiling identifies frequent BRCA2 mutations in MSI-H tumors

**Figure 1:**
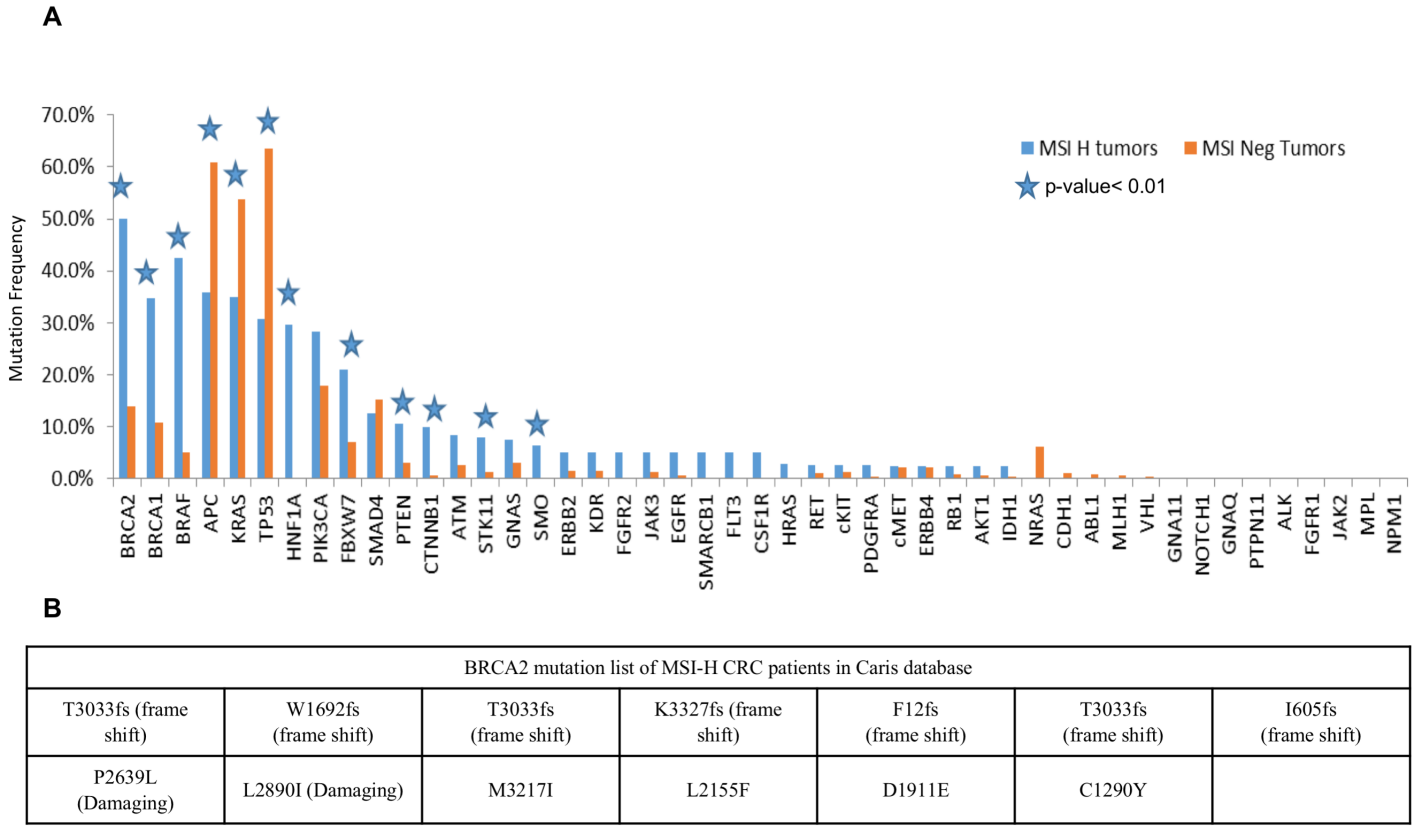
BRCA2 is highly mutated in MSI-H CRCs in a discovery cohort of CRCs profiled by Caris Life Sciences **A**. Selected genes including deregulated genes in CRC in MSI-H and non-MSI-H CRC subtypes is plotted with different mutation frequencies among the MSI-H and non-MSI-H groups are shown. The *p*-value< 0.01 shows the significance of the comparison, represented by stars. **B**. Damaging mutations including frameshift and missense mutations in MSI-H CRCs from the Caris dataset are listed in the table.

We analyzed the mutation data for 26 MSI-H and 558 non-MSI-H CRCs that were profiled at Caris Life Sciences. The MSI-H CRCs showed a significantly higher mutation frequency in the BRCA2 gene as compared to the non-MSI-H tumors (50% vs 14%, P<0.0001) (Figure 1). In the Caris cohort (Figure 1), there was enrichment for BRCA2, BRCA1, and BRAF mutations in MSI-H CRCs while APC, KRAS, and p53 mutations appeared significantly reduced. Additional rarely mutated genes appeared to be significantly increased in MSI-H tumors in the Caris dataset including HNF1A, FBXW7, PTEN, CTNNB1, STK11, and SMO (Figure 1). The specific deleterious BRCA2 mutations found in the Caris dataset in MSI-H CRCs are listed in Figure 1B. Among the frameshift BRCA2 mutations in MSI-H CRCs, 4 (50%) were found in repetitive sequences of the BRCA2 gene. Common MSI-associated mutations in other genes detected in the Caris life sciences dataset include FLCN (H429fs), HNF1A (P291fs), PTEN (K267fs, N323fs, T319fs), RNF43 (G659fs), and MSH6 (F1088fs—occurs in tumors that are already MSI).

We further derived mutation data from the Catalog of Somatic Mutations in Cancer (COSMIC) database and The Cancer Genome Atlas (TCGA) data set [8, 34]. To profile and assess the potentially destructive mutations in BRCA2, we evaluated 101 MSI-H and 916 non-MSI-H profiled samples with various prediction algorithms for their BRCA2 functionally important somatic mutations (Table 1).

**Table 1:** Molecular characteristics of defined cohorts from different databases

			MSI-H CRCs	non-MSI-H CRCs
Caris Life Sciences	Samples		**26**	**558**
	Mutations	BRCA1	5 patients (Neutral)	28 Patients (Neutral)
		BRCA2	13 patients (8/14 frameshift and 2/14 missense damaging mutations)	79 patients
COSMIC (including TCGA data)	Samples		**101**	**916**
	Mutations	BRCA1	28 patients (9/43 damaging mutations)	45 patients (4/48 damaging mutations)
		BRCA2	46 patients (48/75 damaging mutations)	58 patients (25/58 damaging mutation)
		EGFR	46 patients (32/75 damaging mutations targeting TK domain)	60 patients (30/80 damaging mutations targeting TK domain)
		TP53	68 patients (6/137 damaging mutations targeting hotspots)	542 patients (194/606 damaging mutations targeting hotspots)
		POLέ	43 patients (25 damaging)	39 patients (21 damaging)

### Somatic alterations within MLH1 and MSH2 proteins found in MSI-H patient tumors

The COSMIC v73 dataset has profiled patients with cancers including patients with cancer in their large intestine, displaying sequenced genes with mutations [35]. The mutation collections for the large intestine in the present study were derived from the COSMIC whole genome version 73. Analyzing exome sequences, the COSMIC whole genome database profiled 1104 samples with CRCs for their somatic mutations in coding exons [8]. Since microsatellite status (MS) is not available in COSMIC, we designed a cohort with bioinformatics tools to define the MS status regarding the potential loss of function in MMR proteins. We defined coding variations with functional impact on corresponding protein as pathogenic or benign as predicted by the Functional Analysis through Hidden Markov Models (FATHMM) online server (http://fathmm.biocompute.org.uk/). PolyPhen-2 (http://genetics.bwh.harvard.edu/pph2/) was applied to further verify damaging effect of missense mutations in MMR proteins.

**Figure 2:**
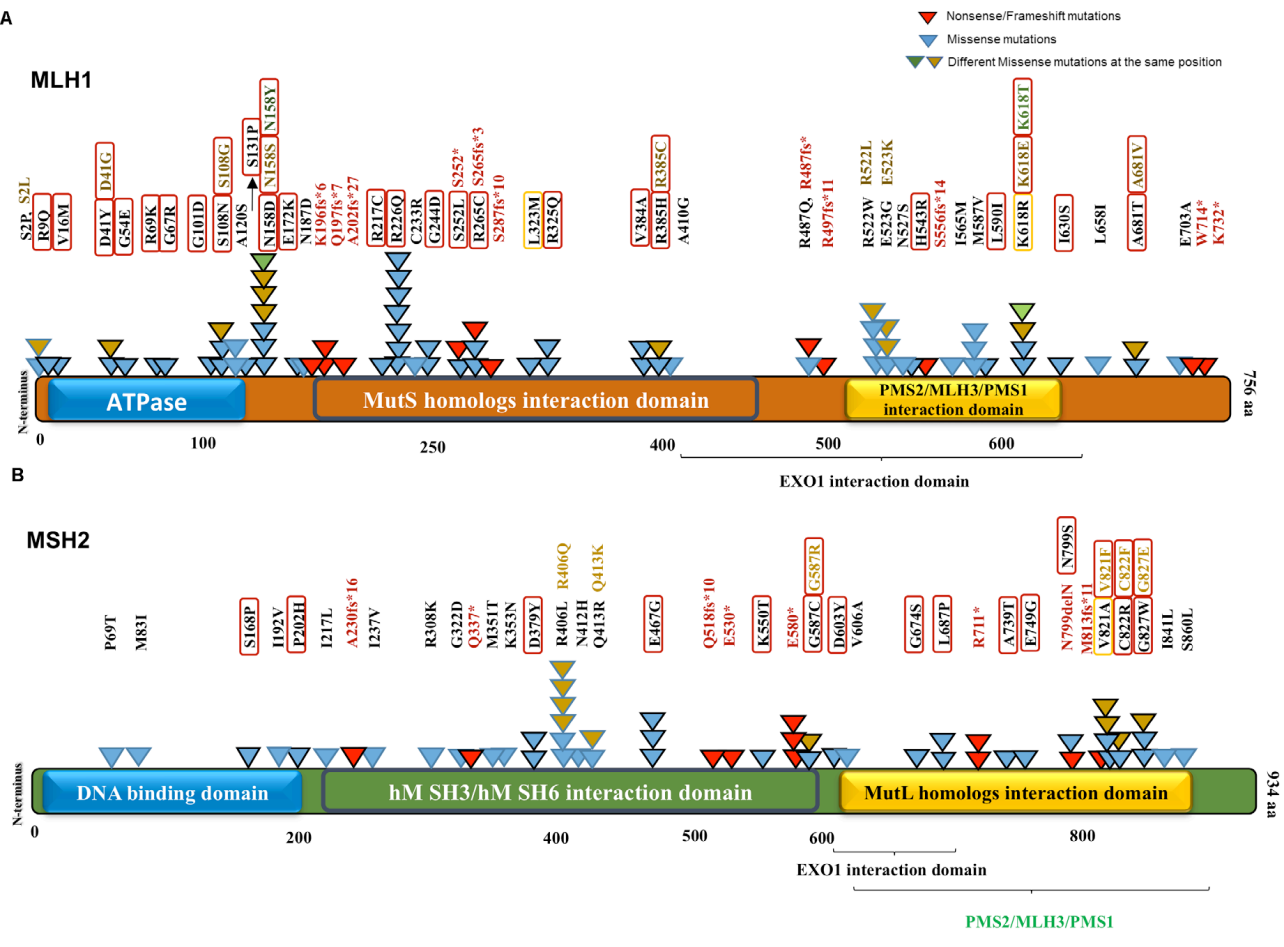
MLH1 and MSH2 protein domains annotated with somatic non-synonymous alterations observed in MSI-H CRCs from the COSMIC database **A**. MLH1 domains and variants. Three known domains are shown in the protein structure. The alterations including missense, nonsense and frameshift mutations are mapped with respect to known domains. Different numbers and colors of triangles in the same positions are representatives of frequency and diversity of mutations in the same spot, respectively. Red triangles represent the truncated mutations, while missense variants are shown in blue, brown and green. Variants predicted by PolyPhen-2 (http://genetics.bwh.harvard.edu/pph2/) to be damaging are denoted in black-outlined triangle as well as red/orange rectangle. Red rectangles are representative of damaging mutations with high probability (>90%) and orange rectangles outline the mutations with possibility (>50%). **B**. MSH2 domains and variants.

The MSI-H cohort was defined based on the presence of damaging mutations in MLH1 and MSH2. CRCs containing either wild-type or synonymous mutated MMR genes MLH1, MSH2, MSH3, MSH6, PMS1 and PMS2 were included in the non-MSI-H (MSS) cohort. Among the profiled CRC samples in the COSMIC version 73, 101 MSI-H and 916 non-MSI-H were categorized according to our definition, i.e. we are comparing MSH2/MLH1-mutant CRC with non-MMR gene mutated tumors. The MSI-L samples with no clear connection with defective MMR genes were excluded from our statistical analysis. Most of the mutations predicted to be pathogenic by FATHMM were also predicted to be damaging via the PolyPhen-2 algorithm, mapped on both MLH1 and MSH2 protein sequences and structural domains (Figure 2). Identified MSH2/MLH1-mutant CRCs exhibited at least one somatic mutation with a damaging effect in MLH1 or MSH2 proteins in some cases was accompanied by other non-synonymous mutation(s) of MMR genes. The frequency of predicted MSH2/MLH1-mutant CRCs in the CRC population under study was observed at 17 percent (Supplementary Figure 1), similar to the reported prevalence of MSI in CRC [7]. As expected, CRC patients with MSH2/MLH1-mutant CRCs are detected more frequently among stage II and less frequently in stage IV (Supplementary Figure 1) [7]. Moreover, POLɛ mutation frequency was significantly increased in MSH2/MLH1-mutant CRCs (MSI-H) (42% vs. 4% in MSS, P<0.0000001) (Table 1), as expected in hypermutable tumors [8].

### In a validation cohort from COSMIC, BRCA2 is among the highly mutated genes in patients with MSH2/MLH1-mutant CRCs

**Figure 3:**
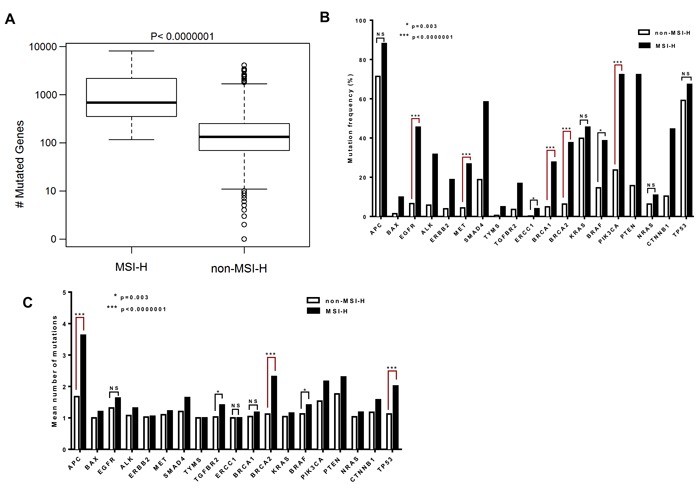
The BRCA2 gene is among the most highly mutated genes with a higher mean number of mutations per tumor in MSI-H CRCs **A**. Difference in number of mutated genes in CRC patients with MSI-H and non-MSI-H are plotted in box-plot (mutation counts are log10 scaled). **B**. Genes with different mutation frequencies among the MSI-H and non-MSI-H groups are shown with respect to matters of significance. **C**. The columns represent the mean of the number of mutations in the gene, across both MSI-H and non-MSI-H samples. The distribution of mutation counts between MSI-H and non-MSI-H samples were compared by Wilcoxon rank sum tests. Fisher exact test was applied to compare the categorical variables.

Microsatellite testing provides a predictive marker to identify the underlying MMR mutations and this may correlate with the mutation rate in the cell [10]. We assessed the number of mutated genes in MSH2/MLH1-mutant CRCs (predicted MSI-H CRCs) versus predicted non-MSI-H tumors to determine whether MSI affects the number of mutated genes. A significantly higher rate of mutated genes in MSH2/MLH1-mutant (MSI-H) CRCs vs. non-MSI-H (median 526 vs 101; P<0.0000001) was found corresponding to non-functional MLH1 and MSH2 proteins (Figure 3A). We identified a 7.4-fold increase in somatic non-synonymous variations in MSH2/MLH1-mutant CRCs compared to non-MSI-H colorectal cancers. A high number of mutated genes in some patients in the non-MSI-H group, shown in Figure 3A could correspond to the chromosomal instability (CIN) pathway as a distinct form of genomic instability promoting CRC [3, 4]. Furthermore, enrichment of POLɛ and EXO1 mutations were detected in 12/33 (36%) non-MSI-H patients with at least 1000 mutations. POLɛ mutations in the absence of MMR-deficiency could lead to a hypermutable phenotype in CRC and do not directly demonstrate microsatellite instability [39]. All the COSMIC variants analyzed came from samples tagged as positive for “genome-wide screen” indicating whole exome sequencing [8]. However, the low number of mutations in patients with less than 10 mutated genes in the non-MSI-H group can be either because of low-quality genome-wide exome sequencing to detect somatic mutations or because possibly only selected genes were sequenced such as APC, KRAS, and TP53 in some tumors (Figure 3A).

Deregulation of RTK-RAS, WNT, PI3K, TGF-β and p53 signaling pathways in CRC has been reported in the Cancer Genome Atlas Network (TCGA) study [8]. To further identify MSI effects on mutation frequency of altered pathways in CRC, we compared the non-synonymous mutation frequency in the MSH2/MLH1-mutant CRCs (MSI-H) versus the non-MSI-H group lacking identified mutations in MMR genes. In this analysis, we identified APC, KRAS, NRAS and p53 to be mutated in both MSH2/MLH1-mutant CRCs (predicted MSI-H) and the non-MSI-H CRC samples with no statistically significant difference: APC (88.11% vs. 71.39%), KRAS (42.57% vs. 39.62%), p53 (62.37% vs. 58. 29%) and NRAS (8.92% vs. 5.78%;) (Figure 3B).

Despite the significantly higher level of APC transcript in tumors predicted as MSI-H, CRC subtypes were frequently mutated for APC with a non-significant difference (Figure 3B, Supplementary Figure 3). Alterations in the p53 pathway were previously found in 59% of non-hypermutated cases, similar to our initial Caris cohort (63%) and the COSMIC cohort (67%) [8]. However, our observation in the COSMIC cohort is indicative of a non-significant difference in TP53 mutated cases among CRC MSI-H or non-MSI-H subtypes (Figure 3B). Both MSI-H and non-MSI-H groups appear to express similar levels of TP53 mRNA (Supplementary Figure 3). Our mutation analysis displays non-MSI-H patients as having a higher frequency of damaging mutations in p53 hotspots [40] at 32% including p.R248, p.G245, p.G244, p.C238, p.M237, p.S215, p.R213, p.Y205, p.R196, p.I195, p.L194, and p.R175H, compared to MSI-H patients at 4%. The data highlights the correlation of p53 mutation and advanced stage of colorectal cancer and the reverse association with MSI [41].

We observed significant enrichment of mutations in cancer-related genes involved in PI3K and TGFβ signaling pathways as well as BRCA genes in MSI-H tumors (Figure 3B). We note that these mutated genes (PI3K, TGFβ and BRCA) have the same level of transcript expression in both MSI-H and non-MSI-H CRC cohorts. CRC patients with BRCA2 mutations are significantly more common in the MSI-H than in the non-MSI-H cohort (37.6% vs 6.3%, p<0.0000001), and the same trend for BRCA1 mutations (27.7% vs 4.9%, p<0.0000001) (Figure 3B). Although KRAS and NRAS are mutated with a high frequency in both MSH2/MLH1-mutant (MSI-H) and non-MSI-H groups, BRAF is more frequently mutated in MSH2/MLH1-mutant CRCs (MSI-H) than in non-MSI-H CRCs (32.67% vs 13.10%; p=0.001) (Figure 3B), consistent with the known association between MSI-H CRCs with BRAF mutations [8, 42].

By contrast, we could not detect a significant difference in mutation frequency among randomly picked housekeeping genes in both the MSH2/MLH1-mutant MSI-H and non-MSI-H cohorts (Supplementary Figure 2). Brain-specific genes are less frequently mutated in both groups compared to the highly mutated list of genes. However, some brain-specific genes such as NGR3 and HAPLN2 were highly mutated in the MSI-H group (Supplementary Figure 2).

A diverse number of mutations was observed in individual CRC patients in each subtype. The distribution of the number of mutations in each gene among MSI-H and non-MSI-H samples with at least one mutation in the particular gene was examined (Figure 3C). The mean number of mutations in BRCA2 was determined to be significantly higher in the MSI-H than the non-MSI-H CRCs (2.3 vs. 1.1, p<0.0001), suggesting that each CRC patient with MSI has a higher number of BRCA2 mutations than the patients in the non-MSI-H subtype (Figure 3C). Although CRC patients in both subtypes have a similar mutation frequency of APC and TP53 in the COSMIC dataset, MSI-H CRCs were observed to harbor more APC and TP53 mutations per patient sample as compared to patients in the non-MSI-H cohort (Figure 3C).

### BRCA2 mutations are distinct between non-MSI-H versus MSH2/MLH1-mutant (MSI-H) CRCs

We investigated BRCA1/2 mutations in the MSH2/MLH1-mutant (MSI-H) and the non-MSI-H CRC patient sample groups to decipher the statistical, structural, and functional difference in BRCA mutations of each group. Among 101 MSH2/MLH1-mutant CRC (MSI-H) patients, 46 CRC patients had 88 (75 unique) somatic BRCA2 mutations including 9 frameshift/nonsense mutations (truncating the protein), 56 missense mutations, and 9 silent mutations along with one mutation in a splicing site (Table 1, Supplementary Figure 4). By contrast, only 58 CRC patients were found to hold 65 (58 unique) somatic BRCA2 mutations among the 916 non-MSI-H patient CRC samples.

**Figure 4:**
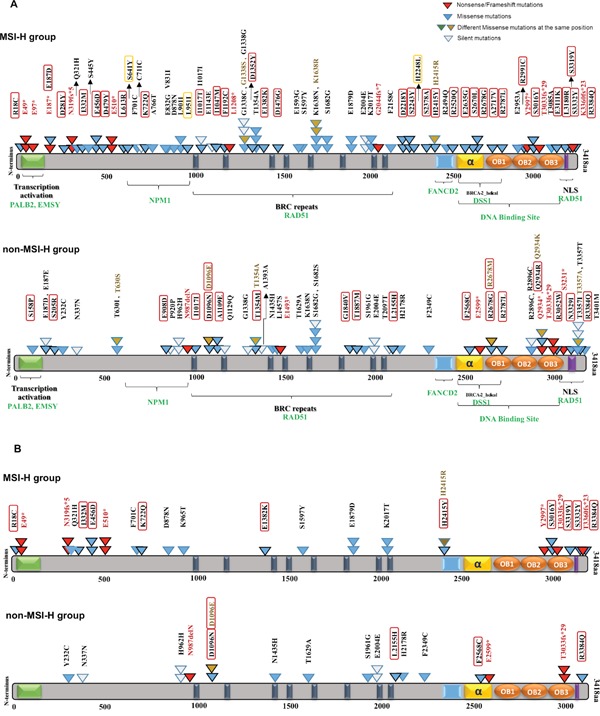
BRCA2 protein domain structure annotated with somatic alterations in MSI-H vs **non-MSI-H CRCs and coding microsatellites**. **A**. BRCA2 mutations in MSI-H patient samples vs. non-MSI-H samples. Functional domains and interaction partner proteins are annotated in black and green, respectively. Truncating variants including nonsense and frameshift mutations are shown in red. Missense mutations are denoted in blue as well as brown in the same spot. Variants predicted by PolyPhen-2 (http://genetics.bwh.harvard.edu/pph2/) to be damaging are denoted in black-outlined triangle as well as red/orange rectangle. Red rectangles are representative of damaging mutations with high probability (>90%) and orange rectangles outline the mutations with possibility (>50%). Synonymous variants are shown in white triangles. **B**. BRCA2 and mutations in coding repetitive sequences in both groups.

Somatic mutations in BRCA2 derived from the COSMIC dataset v73 were mapped on the BRCA2 protein structure with known functional domains (Figure 4). The majority of somatic mutations were missense variants with an unknown functional effect. Using the PolyPhen-2 predictor of protein loss-of-function, we assigned missense mutations as damaging or neutral. A high Polyphen-2 score indicates missense mutations predicted to lead to loss of structural integrity or protein function. Of the 56 BRCA2 missense mutations detected in the MSH2/MLH1-mutant (MSI-H) tumors, 39 (70%) were predicted to be damaging, disrupting protein structure or function (Figure 3, Table 2). We did not detect a significant difference in BRCA1 somatic mutations between the MSH2/MLH1-mutant (MSI-H) vs the non-MSI-H CRC groups (Table 1). Therefore, our study focused more on BRCA2 somatic mutations in MSH2/MLH1-mutant MSI-H CRCs.

**Table 2:** The consensus prediction results of BRCA2 mutations in MSI-H patients

Mutation CDS	Mutation AA	Breast/ovarian/pancreatic/ uterine cancer germline mutations	Consensus	EXAC frequency	Site subtype	Microsatellite target
c.10151G>A	p.R3384Q	BREAST	Deleterious	N/A	rectum	X
c.1334C>A	p.S445Y	N	Neutral	N/A	colon	
c.1368G>T	p.E456D	N	Deleterious	N/A	rectum	X
c.1435G>T	p.D479Y	PANCREATIC	Deleterious	N/A	colon	
c.1838T>G	p.L613R	N	Deleterious	<1/10,000	colon	
c.1922C>A	p.S641Y	N	Deleterious	N/A	caecum	
c.2102T>G	p.F701C	N	Deleterious	N/A	colon	X
c.2164A>C	p.K722Q	N	Deleterious	N/A	rectum	X
c.2296G>A	p.A766T	N	Neutral	N/A	colon	
c.2491G>A	p.V831I	BREAST	Neutral	N/A	colon	
c.2495A>G	p.E832G	N	Neutral	N/A	colon	
c.2632G>A	p.D878N	N	Neutral	<1/10,000	caecum	X
c.2701C>A	p.L901I	N	Neutral	N/A	colon	
c.2851C>A	p.L951I	N	Neutral	N/A	colon	
c.2867A>C	p.K956T	N	Neutral	N/A	colon	
c.3050T>C	p.I1017T	SAME POSITION	Deleterious	N/A	colon	
c.3141T>G	p.I1047M	N	Deleterious	N/A	rectum	
c.3575T>G	p.F1192C	BREAST	Deleterious	1/10,000-0.001	colon	
c.4012G>A	p.G1338S	SAME POSITION	Deleterious	N/A	caecum	
c.4012G>T	p.G1338C	SAME POSITION	Deleterious	N/A	colon	
c.4054G>T	p.D1352Y	BREAST	Deleterious	N/A	colon	
c.4144G>A	p.E1382K	SAME POSITION	Deleterious	N/A	colon	X
c.4427A>G	p.D1476G	N	Deleterious	Singleton	colon	
c.4778A>G	p.E1593G	N	Deleterious	N/A	colon	
c.4790C>A	p.S1597Y	N	Neutral	N/A	rectum	X
c.4913A>G	p.K1638R	OVARIAN	Neutral	N/A	caecum	
c.4914A>T	p.K1638N	N	Deleterious	N/A	caecum	
c.52C>T	p.R18C	N	Deleterious	N/A	colon	X
c.561G>T	p.E187D	SAME POSITION	Deleterious	Singleton	caecum	
c.5637G>T	p.E1879D	SAME POSITION	Deleterious	N/A	rectum	X
c.6050A>C	p.K2017T	N	Deleterious	N/A	rectum	X
c.6473T>G	p.F2158C	N	Neutral	N/A	caecum	
c.6652G>T	p.D2218Y	N	Deleterious	N/A	rectum	
c.6728C>A	p.S2243Y	N	Deleterious	N/A	rectum	
c.6743A>T	p.H2248L	N	Neutral	N/A	NS	
c.7132T>G	p.S2378A	SAME POSITION	Deleterious	N/A	rectum	
c.7243C>T	p.H2415Y	N	Deleterious	N/A	colon	X
c.7244A>G	p.H2415R	N	Neutral	Singleton	colon	X
c.7481G>A	p.R2494Q	BREAST	Deleterious	N/A	rectum	
c.7559G>A	p.R2520Q	BREAST	Deleterious	<1/10,000	caecum	
c.7904A>G	p.E2635G	N	Deleterious	N/A	colon	
c.8009C>T	p.S2670L	BREAST	Deleterious	N/A	rectum	
c.8032A>G	p.R2678G	N	Deleterious	N/A	colon	
c.8150C>T	p.A2717V	SAME POSITION	Deleterious	N/A	colon	
c.8360G>T	p.R2787L	SAME POSITION	Deleterious	N/A	caecum	
c.841G>T	p.D281Y	SAME POSITION	Deleterious	N/A	colon	
c.8858A>C	p.E2953A	N	Neutral	N/A	caecum	
c.8971C>T	p.R2991C	SAME POSITION	Deleterious	Singleton	colon	
c.9047C>A	p.S3016Y	N	Deleterious	N/A	rectum	
c.9253A>G	p.T3085A	SAME POSITION	Neutral	N/A	caecum	X
c.9331G>A	p.E3111K	N	Deleterious	N/A	colon	
c.9539T>G	p.L3180R	SAME POSITION	Deleterious	N/A	rectum	
c.963A>C	p.Q321H	SAME POSITION	Neutral	N/A	rectum	X
c.9956C>A	p.S3319Y	N	Deleterious	N/A	colon	X
c.996T>G	p.I332M	N	Neutral	N/A	caecum	X
c.9995C>A	p.S3332Y	N	Deleterious	N/A	colon	X

We show that MSH2/MLH1-mutant (MSI-H) CRCs display a distinct pattern of BRCA2 mutations as reflected in the frequency, diversity and position of the mutations by comparison to the non-(MSI-H) CRCs. Significantly more BRCA2 mutations were predicted to be damaging in the MSH2/MLH1-mutant MSI-H CRCs as compared to the non-MSI-H CRCs (64% vs 43%, p=0.0045) (Figure 4). More frameshift and/or nonsense point mutations were observed to be distributed in the N-terminal terminal region of BRCA2 in the MSH2/MLH1-mutant MSI-H group (Figure 4), leading to severely truncated protein and deficient HR in the cell. Most of the observed mutations accumulated in the C-terminal region of BRCA2 targeting microsatellites and highlighting the functional importance of this area, through interaction with DSS1 and RAD51 to facilitate HR [43]. Only few of the BRCA2 mutations in CRCs have been reported in breast/ovarian cancer suggesting that these mutations may be CRC-specific.

Different repetitive sequences (mononucleotides, dinucleotides, and trinucleotides) and their frequency were sought in BRCA2 and they were integrated with corresponding somatic mutations and involved domains. Of 123 distinct BRCA alterations in both CRC groups, 39 variations with a deleterious effect were mapped on coding microsatellites (Supplementary Table 1, Figure 4B). Altogether, our data suggest that coding microsatellites in BRCA2 are more mutated with a higher potential for damaging mutations in the MSI-H patients than in the non-MSI-H group. This analysis highlights the significance of the underlying genetic signature and potential impact of deficient MMR on mutations of coding microsatellites in BRCA2.

### Functional prediction modeling reveals candidate damaging BRCA2 mutations

**Figure 5:**
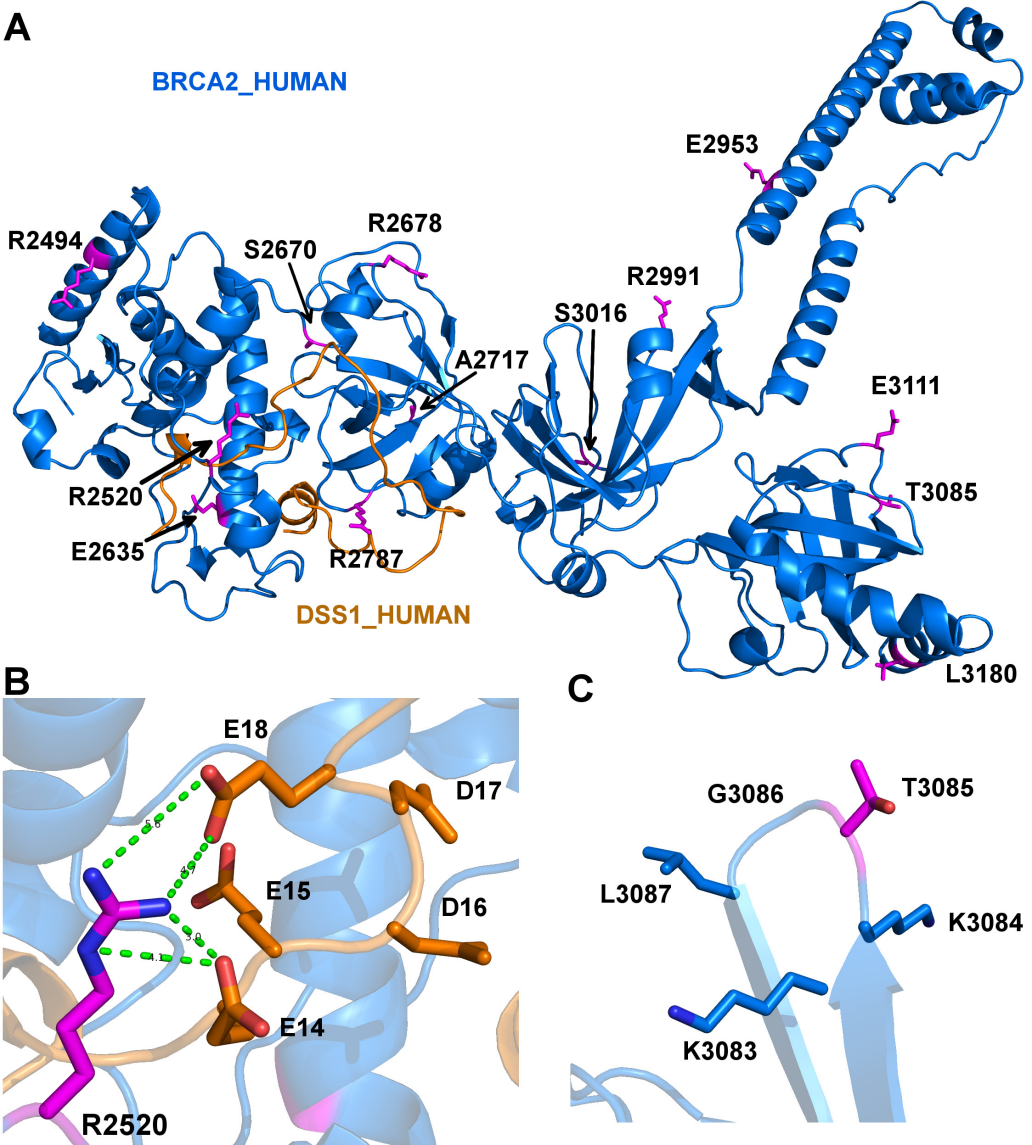
Model depicting the mutations in human BRCA2 that may impact interaction with DSS1 **A**. The structure contains 13 mutations which are colored in magenta and shown in sticks. The residues and their sequence numbers are labeled in black. The figure is generated by PyMOL (https://www.pymol.org/pymol). **B**. The hydrogen bonds of Arg2520 and Glu14 and Glu18 of DSS1. The hydrogen bond is colored in green and shown in dot lines. The residues are colored in elements, where oxygen atom is colored in red, nitrogen is colored in blue. A hydrophilic pocket of DSS1 is composed of all negative electrically charged residues Glu14, Glu15, Asp16, Asp17 and Glu18. **C**. Thr3085 has no side-chain interactions with its neighbor residues (Lys3083, Lys3084, Gly3086 and Leu3087).

We sought to identify somatic mutations that may damage protein function. We used five different algorithms to predict BRCA2 mutations for their destructive effects on the encoded protein, including PolyPhen-2 (Polymorphism Phenotyping v2) [44], SIFT (Sorting Intolerant From Tolerant) [45], PROVEAN (Protein Variation Effect Analyzer) [46], MutPred [47], and a predictor using support vector machine (SVM) developed by Wei and Dunbrack, based on structural analysis of protein complexes [48]. We used the consensus result from these five predictors. A mutation is predicted to be deleterious if at least three predictors designated it to be deleterious. Structural information was used to verify the predictions. Since there is no human BRCA2 structure containing our mutations available in the Protein Data Bank (PDB), we used a mouse BRCA2 structure PDB: 1MIU as our template to model human BRCA2 [49]. 1MIU is a complex structure consisting of a mouse BRCA2 chain (sequence [2378–3115]) and human DSS1 proteins (Figure 5A).

Table 2 shows the consensus result from functional predictors and structural modeling. We further searched for common mutations of BRCA2 in breast and ovarian cancers in the Breast Cancer Information Core (BIC) (http://research.nhgri.nih.gov/bic/), and pancreatic and uterine cancer in COSMIC. Among all missense mutations of BRCA2 in MSH2/MLH1-mutant MSI-H CRCs, 9 were previously reported in breast, ovarian, pancreatic and uterine cancers and 14 variants involved the same reported sites in breast cancer but with different amino acid substitutions in CRC. Except for 3, the other 20 mutation hits with history of occurrence in different types of cancer including breast and ovarian have damaging effect on BRCA2 protein (Table 2).

### EGFR is highly mutated in MSH2/MLH1-mutant MSI-H CRCs targeting the tyrosine kinase (TK) domain

**Figure 6:**
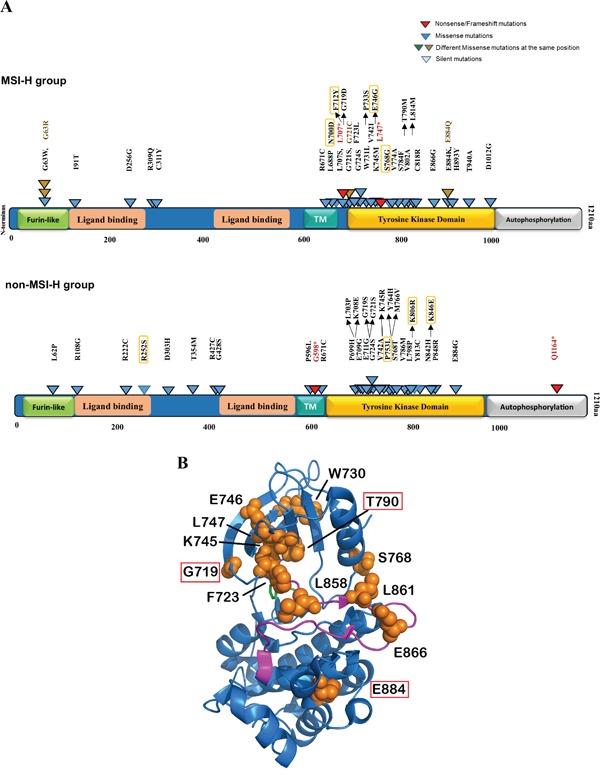
EGFR mutations in kinase domain denoted on 2D and 3D model of EGFR protein structure **A**. EGFR protein domain structure are annotated with activating as well as potentially damaging somatic alterations in MSI-H vs. non-MSI-H CRCs. Functional domains are annotated in different colored-boxes. Truncating variants including nonsense and frameshift mutations are shown in red. Missense mutations are denoted in blue as well as brown in the same spot. Variants predicted by PolyPhen-2 (http://genetics.bwh.harvard.edu/pph2/) to be damaging are denoted in black-outlined triangle. Orange rectangles outline the mutations with possibility (>50%). **B**. Model depicts mutations in tyrosine kinase domain of EGFR genes. The structure of EGFR kinase domain is showing the WT residues of mutations in orange spheres. The activation loop is colored in magenta and Phenylaniline residue of the DFG motif is shown in green sticks. The known responding mutations to TKI are denoted in red outlined. The figure is generated by PyMOL (https://www.pymol.org/pymol).

Unlike the previous reports (21, 25), our mutation analysis of the COSMIC dataset revealed a significantly higher EGFR mutation frequency in the MSH2/MLH1-mutant CRC (MSI-H) subtype (45.5% vs. 6.5% in non-MSI-H CRCs, p<0.0000001) (Figure 3B). To determine the domain distribution and functional patterns of EGFR mutations in CRC, we mapped the deleterious-predicted mutations including nonsense and missense mutations. Of 101 MSH2/MLH1-mutant MSI-H CRCs, 46 patients had 75 EGFR mutations including 2 nonsense mutations (with inactivating effect), 53 missense mutations, and 20 silent mutations (Table 1). Somatic EGFR missense mutations derived from the COSMIC database v73 were predicted by PolyPhen-2 and FATHMM for their deleterious/dysfunctional effects, mapped on the EGFR protein structure with respect to known domains (Figure 6). Of 53 missense mutations, 34 (64%) were predicted to have damaging effect on EGFR protein structure (Figure 6, Table 1). A high frequency of EGFR mutations (82%) was observed in the TK domain, targeting exons 18-24. Our sequence-structure alignment, developed by Modi and Dunbrack (unpublished) shows that 50% of EGFR mutations in the TK domain are located in the same spot as known activating mutations in other kinases including AGC, CAMP, tyrosine kinases and tyrosine-like kinases [50](Figure 6B). Approximately 15% of the EGFR mutations found in the TK domain are known activating mutations and may therefore be actionable through TKI therapy, including the N700D, G719D, T790M, and E884K EGFR mutants. Of note, the most common EGFR mutation (L858R) observed in lung cancer was not found on the MSI-H CRC COSMIC dataset. The observed EGFR L747* in MSI-H CRC involves a hotspot that is frequently involved in lung cancer as various small in-frame deletions leading to kinase activation and sensitivity to TKI therapy. However, in MSI-H CRC, L747* results in a stop codon, truncating the protein early in the kinase domain and would not lead to kinase activation or EGFR inhibitor sensitivity. EGFR T725M detected in the Caris cohort of MSI-H CRCs is activating in the absence of EGFR ligand [51]. The observed kinase-activating EGFR mutants retain the extracellular domain with potential for targeted therapy by antibodies or small molecules targeting the TK domain (Figure 6). EGFR protein expression can be regulated transcriptionally (42); however, our analysis found no significant difference in EGFR mRNA expression between the MSI-H and non-MSI-H groups (Supplementary Figure 3).

### NTRK gene mutations occur in MSH2/MLH1-mutated CRCs

**Figure 7:**
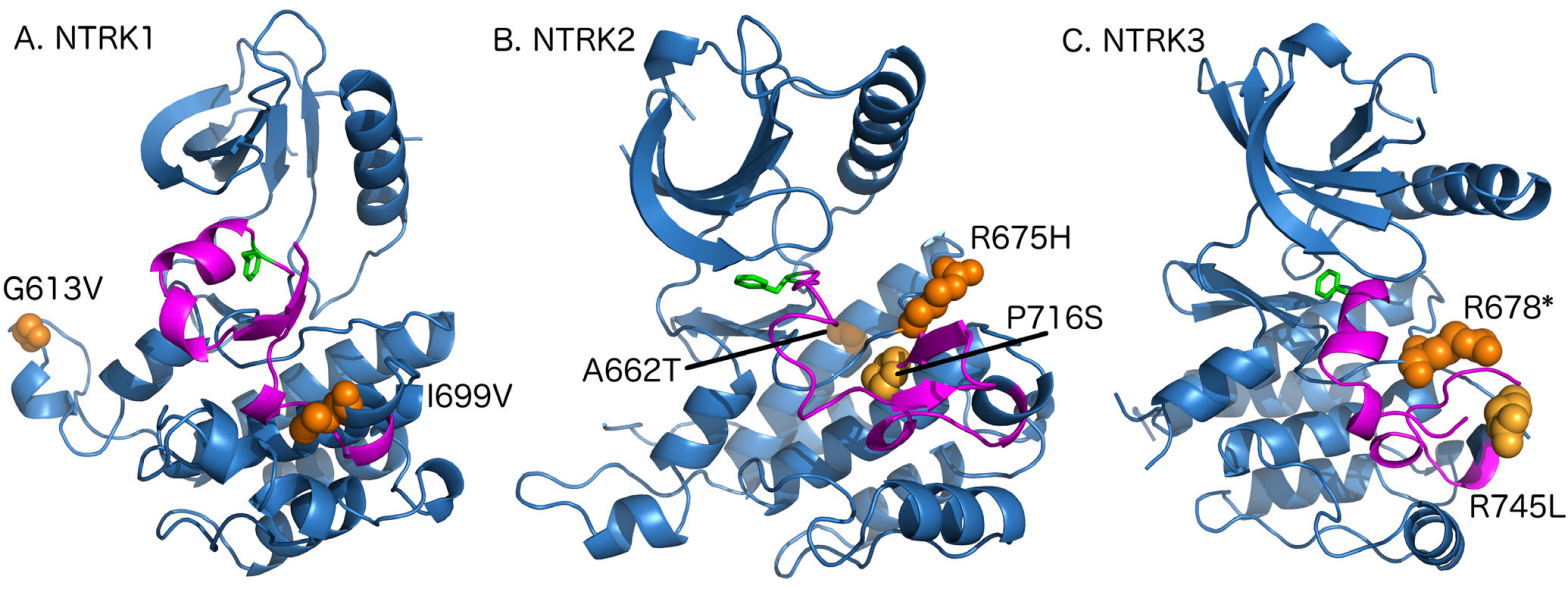
Model depicting the mutations in tyrosine kinase domain of NTRK genes **A**. The structure of NTRK1 kinase domain showing the WT residues of mutations (I613V, I699V) in orange spheres. The activation loop is colored in magenta and Phenylaniline residue of the DFG motif is shown in green sticks. The figure is generated by PyMOL (https://www.pymol.org/pymol). **B**. The structure of NTRK2 kinase domain showing the WT residues of mutations (A662T, R675H, P716S) in orange spheres. **C**. The structure of NTRK3 kinase domain showing the WT residues of mutations (R678*, R745L) in orange spheres.

We searched for mutations in NTRK1/2/3 genes because of the availability of small molecule therapeutic kinase domain inhibitory agents that are currently under investigation in clinical trials [36]. NTRK mutations are rare and we hypothesized they may be enriched in MSI-H CRCs due to increased mutation frequency. Our analysis of TCGA shows that 40% of MSH2/MLH1-mutant CRCs have NTRK mutations versus 16% of non-MSI-H patients (p-value 0.0003) (Supplementary Figure 5). We identified NTRKs mutations in tyrosine kinase domains of NTRK genes including NTRK1 (G613V, I699V), NTRK2 (P716S, R675H, A662T) and NTRK3 (R678*, R745L). These are newly recognized to occur in MSH2/MLH1-mutant (MSI-H predicted) CRCs. G613V (NTRK1) and A662T (NTRK2) are conservative mutations far from the activation loop and also located on the surface of the protein; it is likely that they are neutral mutations. However, R675H (NTRK2) and R678* (NTRK3) are both located at the arginine position of the catalytic HRD motif. Both will inactivate the kinase domain through disrupting the catalytic machinery or producing a truncated protein, respectively. Finally, three of the mutations are either in the activation loop (NTRK2 P716S) or immediately adjacent to it both in sequence and structure (NTRK1 I699V and NTRK3 R745L). They could destabilize the inactive conformation of the kinase domain thereby potentially activating the kinase (Figure 7).

## DISCUSSION

MSI caused by a deficient MMR system leads to the hypermutable phenotype that is detected in ~15% of all CRCs. Each cancer and its subtypes are characterized by a specific somatic mutation signature. Large-scale tumor sequencing studies have produced numerous correlations [52]. However, distinguishing tumor “driver” mutations from “passenger” mutations can be a challenge. Functionally damaging mutations or cancer-driving mutations are usually differentiated from neutral mutations based on their frequency despite the fact that they could occur at very low frequencies among tumors [16].

The lack of microsatellite instability status designation in the COSMIC database is a limitation of our study. Hence, we designed the cohorts based on the presence or absence of common MMR gene mutations associated with the MSI phenotype. CRC patients harboring non-synonymous mutations in MSH3/MSH6/PMS1/PMS2 genes were designated as MSI-L. MSI-L samples that are typically associated with a weak MMR-deficiency phenotype were neither included in the non-MSI-H group nor statistically analyzed in our study. Less than 4 percent of non-MSI-H patients in the COSMIC database were noted to have >1000 mutated genes, in which 36% harbored POLɛ and EXO1 mutations. These were excluded from analysis of non-MSI-H tumors because they were not expected to have microsatellite instability.

The BRCA2 protein is a fundamental element of HR and somatic mutations in BRCA2 are known cancer drivers [15, 16]. The high frequency of repetitive sequences in BRCA2 could allow for frequent mutations in MSI tumors [19]. Our data show a distinct pattern and comparatively frequent BRCA2 mutations in MSI-H CRCs. Functionally damaging mutations predicted in BRCA2 may disrupt protein-protein interactions (Figure 3A, Table 2). Homologous recombination is defective in cancer cells with mutant BRCA1 or BRCA2 genes, leading to more genetic abnormalities. Our results represent the accumulation of relatively CRC-exclusive BRCA2 mutations in the C-terminal area where BRCA2 interacts with DSS1 and RAD51 to facilitate HR. Yet to be further studied, discriminating the environmental influence on somatic mutations as tumor-specific ones may be pursued. Dysfunctional or semi-functional BRCA2 can affect drug sensitivity, especially if biallelic. Likewise, we show that mutations in the BRC repeats of BRCA2 represent another hotspot, contributing to partial functionality or even protease-mediated degradation. It is clear based on present knowledge that tumors with biallelic loss of BRCA2 may be considered for therapeutic strategies that use PARP inhibitors. The unavailability of allele frequency in the COSMIC database particularly for BRCA2 mutations is reflective of another limitation to our study.

EGFR has an extracellular ligand-binding domain, a single membrane-spanning region, and a cytoplasmic tyrosine kinase domain. Activation of EGFR by a ligand leads to phosphorylation of its cytoplasmic tail. EGFR activation stimulates complex intracellular signaling pathways such as RAS-RAF-MAPK and PTEN-PI3K-AKT pathways. A group [53] previously reported a frequency of 22.4% of EGFR mutations targeting exon 18 in CRC patients in Korea. Our mutational analysis of the COSMIC dataset indicates a 45.5% EGFR mutation frequency in CRC patients with MLH1/MSH2 deficient (MSI-H) tumors. EGFR mutations detected in CRC mainly target the TK domain, covering exons 18-24, which have potential for therapeutic targeting by anti-EGFR antibodies or small molecule EGFR inhibitors. A small number of nonsense EGFR mutations leading to truncated protein and inactivating effect were detected in CRCs. However, we predicted about 65% of EGFR missense mutations targeting the TK domain have damaging effect on protein structure with potential activating or inactivating consequences (Figure 6, Table 1).

The interaction between proteins and background expression patterns can be utilized in pharmacological studies (45). We did not detect different mRNA expression levels of EGFR mRNA between CRC subtypes. The lack of access to the protein expression level of EGFR in COSMIC database was another limitation of our study. Furthermore, NSCLCs with EGFR mutations and half of CRC patients without KRAS mutation benefit from anti-EGFR therapies. We detected 477 (43%) colorectal cancer patients profiled in COSMIC V73 to have KRAS mutations, reflecting that almost half of CRC patients are not responsive to anti-EGFR antibody therapies. Non-MSI-H CRCs with KRAS mutations, in particular, were highly (80%) mutated in the G12 and G13 positions such as G13V and G13D compared to 14 (26%) MSI CRCs. The data suggests that non-MSI-H patients are more likely to harbor KRAS mutations that make them resistant to anti-EGFR antibodies such as Cetuximab or panitumumab.

The limited response to EGFR therapy may in part be related to BRAF mutations, which are significantly increased in MSI-H CRCs, or possibly due to lack of patient selection, i.e. we may suggest here therapeutic targeting for patients with EGFR mutation as a potential precision medicine approach. EGFR inhibitors targeting tyrosine kinase (TKI) are not known to be effective drugs for CRC patients with KRAS mutation. Our data are indicative of the presence of a patient with T790M mutation before treatment profiled in COSMIC. The EGFR T790M mutation may rarely occur as a primary resistance mutation together with a sensitizing mutation. Although, the T790M mutation in EGFR confers resistance to EGFR TKIs Gefitinib or Erlotinib (Figure 6)(44), Osimertinib, a third-generation EGFR inhibitor, targets the T790M mutation in EGFR [54]. On the other hand, patients with N700D, and G719D have been reported to be sensitive to TKIs and may benefit from Gefitinib or Erlotinib. While L747 in frame deletion mutants are common in lung cancer and are sensitive to EGFR TKIs, the L747* in MSI-H would not be expected to activate the kinase and is predicted to be insensitive to TKIs. The EGFR E884K mutation confers sensitivity to Gefitinib, but resistance to Erlotinib (46). EGFR T725M detected in our discovery cohort MSI-H CRCs (Caris Life Sciences), is reported in cell-based assays to increase EGFR activity in the absence of EGFR ligand [51]. EGFR activating mutations do not necessarily disrupt the folding or stability of the protein but may alter the dynamics of the protein (Figure 6B). Functional prediction of the mutations via 3D modeling tools are helpful but not sufficient. Characterizing the EGFR activating mutations via identifying autophosphorylation of EGFR via immunohistochemistry (IHC) can be done to establish which CRC patients with EGFR-mutated tumors may derive any benefit from EGFR-targeted therapeutics.

Considering the approximate 134,000 new cases of CRC per year in the United States, 13,000-18,000 are expected to be part of an MSI-H cohort and among them, there would be about 6000-9000 patients with EGFR mutations. These patients have potential to benefit from EGFR inhibitors including antibodies such as Cetuximab, Panitumumab or small molecules such as Erlotinib, Gefitinib or Osimertinib. Most of these patients have early stage disease and may not require chemotherapy although the risk of more aggressive disease in tumors that harbor these mutations is unknown. It may be useful to better understand the relationship between EGFR mutations and disease outcome in early stage CRC and to also consider investigating a potential role for EGFR inhibtiors in the adjuvant setting.

It is also important to further investigate whether selected patients with metastatic disease that is MSI-H (who likely represent a smaller subset of 2000-3000 patients per year or ~4% of patients with advanced CRC) who have no remaining therapeutic options, and who may have progressed on immunotherapy, may derive some benefit from TKI therapy. Based on our results, a subset of the patients with MSI-H advanced disease, perhaps a few hundred patients each year in the US, should have actionable kinase activating EGFR mutations that may respond to the various anti-EGFR therapeutics.

Our findings suggest that other oncogenic driver RTKs that may be activated in MSI-H patients. For example, our analysis of TCGA has shown 40% of MSI-H patients have NTRK mutations in comparison to 16% of non-MSI-H patients (p-value 0.0003). Among these NTRK mutations in TCGA, NTRK1 (G613V, I699V), NTRK2 (P716S, R675H, A662T) and NTRK3 (R678*, R745L) are newly recognized to occur in MSI-H CRCs. All of these mutations are within the kinase domains of NTRK1, NTRK2, and NTRK3 but based on their location within the domain may indicate their potential functional effects. Currently, there are potent kinase inhibitors against NTRK mutated cancers being tested in clinical trials which could benefit patients harboring activated NTRK mutants. Further functional studies are required to validate the predictions from molecular modeling.

It is important to note some limitations of our study including the fact that the discovery cohort and second cohort are not identical in the capture of clinical cases with mismatch repair deficiency. This is due to the fact that there is no true MSI testing in the COSMIC cohort. Nonetheless despite this major limitation, in the clinic a mutation in MSH2 or MLH1 in a tumor is generally managed in the same way as a finding of MSI by MSI testing and both contribute to a high tumor mutation burden in CRC. Mechanistically, a deleterious mutation in MSH2 or MLH1 leads to MSI. Thus we believe the two cohorts are comparable as long as the differences are noted. A discrepancy in our results is that the EGFR mutation rate was found to be higher in the COSMIC dataset versus the Caris dataset. The reasons are not entirely clear but could be due to the more limited sample size in the Caris dataset.

Overall, we characterized BRCA2, EGFR, and NTRK mutations in CRC patients, focusing on the mutations that offer insight into pathogenesis and have significant clinical therapy implications. Future studies will need to address the functional consequences of each mutation that was assessed based on predictive modeling. This includes immunohistochemical assessment of downstream signaling within tumors, overexpression studies, potential use of mouse models, and actual testing of mutation-bearing tumor cells for drug sensitivity. In the era of precision medicine, we note that various mutations may be detected through use of liquid biopsy, and it will be of interest to further assess for the presence of such mutations as tumors evolve. Clinical testing of potential targeted therapeutics in specific patients whose tumors harbor specific mutations would be a reasonable path forward when possible. We believe that our results showing frequent BRCA2, EGFR, and NTRK mutations in MSI-H CRC patients, and potentially other cancers with mismatch repair deficiency, offer immediate novel personalized medicine strategies to treat the patients with advanced disease who may have no remaining treatment options.

## MATERIALS AND METHODS

### Microsatellite status determination

Our initial cohort included 26 MSI-H and 558 microsatellite stable (MSS) cases that were profiled at Caris Life Sciences (Phoenix, AZ) using immunohistochemistry (IHC) and sequencing (NextGen and Sanger). MSI status was determined using a combination of IHC (MLH1, PMS2, MSH2, MSH6) and MIA (Microsatellite Instability Analysis) fragment analysis. MIA included fluorescently-labeled primers for co-amplification of seven markers including five mononucleotide repeat markers (BAT-25, BAT26, NR-21, NR24 and MONO-27) and two pentanucleotide repeat markers (Penta C and D). The mononucleotide markers were used for MSI determination while the pentanucleotide markers were used to detect either sample mix-ups or contamination. A sample was considered MSI-H if two or more mononucleotide repeats were abnormal while MSI-L if one mononucleotide repeat was abnormal. The tumors were considered MSS if mononucleotide repeats were identical between the tumor and adjacent normal tissue.

### Bioinformatics workflow

We downloaded the full Cosmic V73 whole-genome data and TCGA [COAD] level 3 expression data. The COSMIC data was filtered by selecting all mutations occurring in the large intestine, excluding mutations flagged as SNPs, and removing duplicate mutations (i.e., identical mutations labeled with different transcripts). Annotation of variants was performed with Annovar (PMID 20601685), including deleteriousness prediction scores such as PolyPhen-2.

Tissue-specific genes are picked and gene expression levels were confirmed with the TIGER (Tissue-specific Gene Expression and Regulation) database.

Breast Cancer Information Core (BIC), an Open Access On-Line Breast Cancer Mutation Data Base, was applied to detect the previous identified BRCA2 mutations.

### Statistical analysis

Differences in sample proportions were compared using Fisher's exact tests. Wilcoxon rank sum tests were used to perform between comparisons of continuous variables including mutation counts between MSI-H and non-MSI-H samples. All statistical tests were 2-sided. Statistical significance was defined as P < 0.01. Bonferroni adjustment for multiple comparisons was done.

### Functional prediction modeling

We used five predictors to predict the consequences of BRCA2 mutations, including PolyPhen-2 (Polymorphism Phenotyping v2) [44], SIFT (Sorting Intolerant From Tolerant) [45], PROVEAN (Protein Variation Effect Analyzer) [46], MutPred [47], and a predictor using support vector machine (SVM) developed by Wei and Dunbrack [48]. These methods are trained on existing sets of mutation/phenotype association data and use sequence information from homologues, structure information, such as accessible surface area, and changes in amino acid properties to provide feature information as input to machine learning methods for phenotype prediction. PolyPhen-2 provides the probability of being deleterious. If the probability is less than 0.5, the mutation is considered to be “benign”, otherwise, it is considered as “probably damaging”. SIFT outputs a normalized probability for each amino acid type, and a value of less than 0.05 is considered deleterious. PROVEAN uses an alignment-based score that measures the variation of a query sequence and its homolog before and after mutation. The cutoff is -2.5 where PROVEAN has best specificity and sensitivity. A value of less than -2.5 is considered deleterious. MutPred provides probabilities of gain or loss of structure and function. We used MutPred values of 0.5 as cutoff with a value of less than 0.5 considered to be deleterious. The SVM predictor used both sequence and structural information, trained on balanced data sets of deleterious and neutral mutations. The SVM predictor also yields probabilities where a value >0.5 is considered as deleterious. We used BioAssemblyModeler (BAM) [55] software to do homology modeling: first backbone atoms are copied from the template structure, then side-chain coordinates are built with the program SCWRL4 [56]. We used YASARA web site [57] [http://www.yasara.org/minimizationserver.htm] to perform energy minimization using the YASARA force field. All BRCA2, EGFR and NTRK structures were studied in PyMOL (https://www.pymol.org/). We assessed the mutations by using FATHMM (http://fathmm.biocompute.org.uk/cancer.html) to distinguish between cancer promoting/drivers and germline polymorphisms.

## SUPPLEMENTARY MATERIALS FIGURES AND TABLES





## Abbreviations

MSI: Microsatellite instability
CRC: Colon Cancer
MMR: Mismatch repair system
HPNCC: hereditary non-polyposis colorectal cancer
DSB: Double strand break
HR: Homologous recombination
TKI: Tyrosine kinase inhibitor
EGFR: Epidermal growth factor
COSMIC: Catalog of Somatic Mutations in Cancer
TCGA: The Cancer Genome Atlas
CIN: Chromosomal instability
PolePhen-2: Polymorphism Phenotyping v2
SIFT: Sorting Intolerant From Tolerant
BIC: Breast Cancer Information Core.
