# Supplementary material for: BRCA2, EGFR, and NTRK mutations in mismatch repair-deficient colorectal cancers with MSH2 or MLH1 mutations
Source: Oncotarget. 2017 May 23;8(25):39945–62. doi: 10.18632/oncotarget.18098 (PMC5522275; doi:10.18632/oncotarget.18098)
Supplement: Supplementary file 1 [file oncotarget-08-39945-s001.pdf]

# BRCA2, EGFR, and NTRK mutations in mismatch repair-deficient colorectal cancers with MSH2 or MLH1 mutations

## Supplementary Material

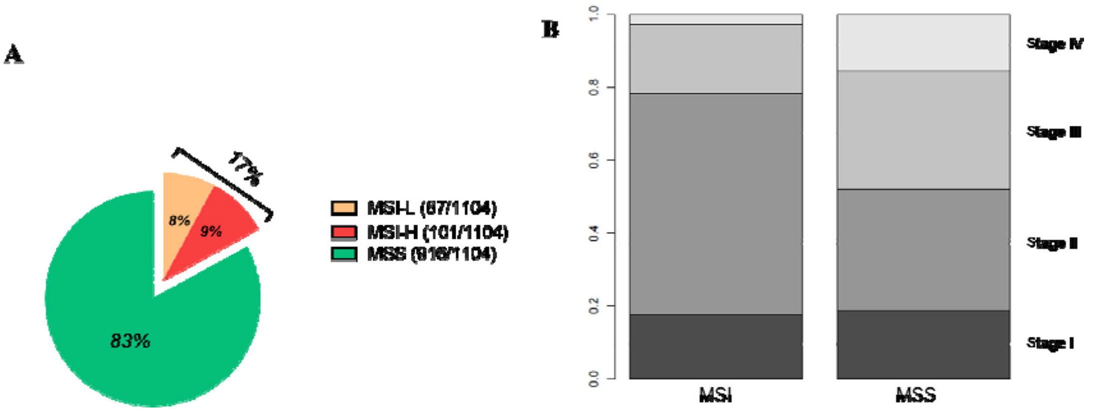


**Supplementary Figure S1.** 17% of CRCs, the predicted MSI cohort, has high frequency of stage II CRC. (A) Distribution of CRC subtypes are plotted in the Pie chart. MSI includes both MSI-H and MSI-L populations. (B) Distribution of CRC stages of both MSI and MSS groups in the cohort is plotted together.

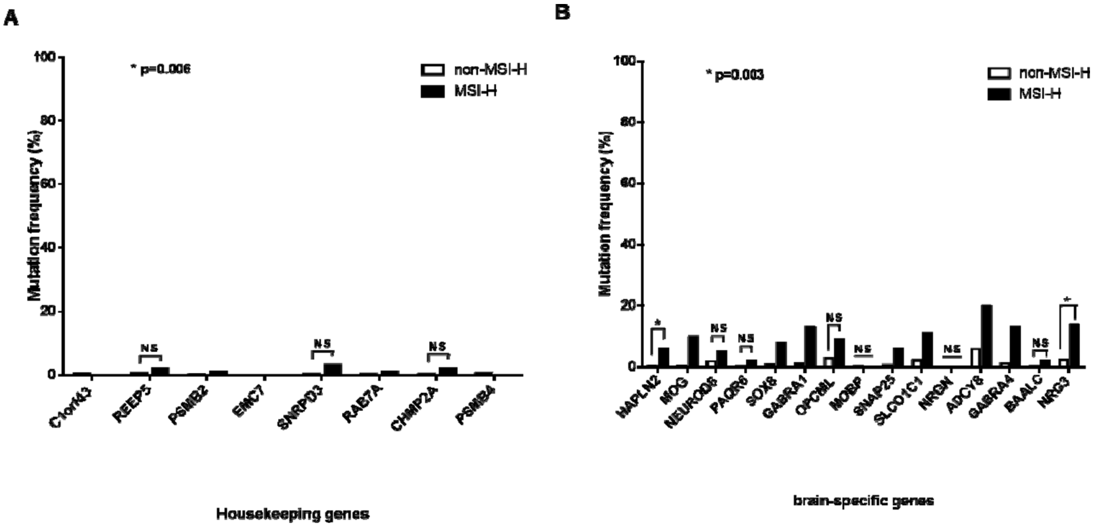


**Supplementary Figure S2.** Low frequency of mutations is seen in either randomly picked housekeeping or brain-specific genes (44). (A,B) Mutation frequencies in MSI-H and non-MSI-H groups are shown with p-values from Fisher's exact tests.

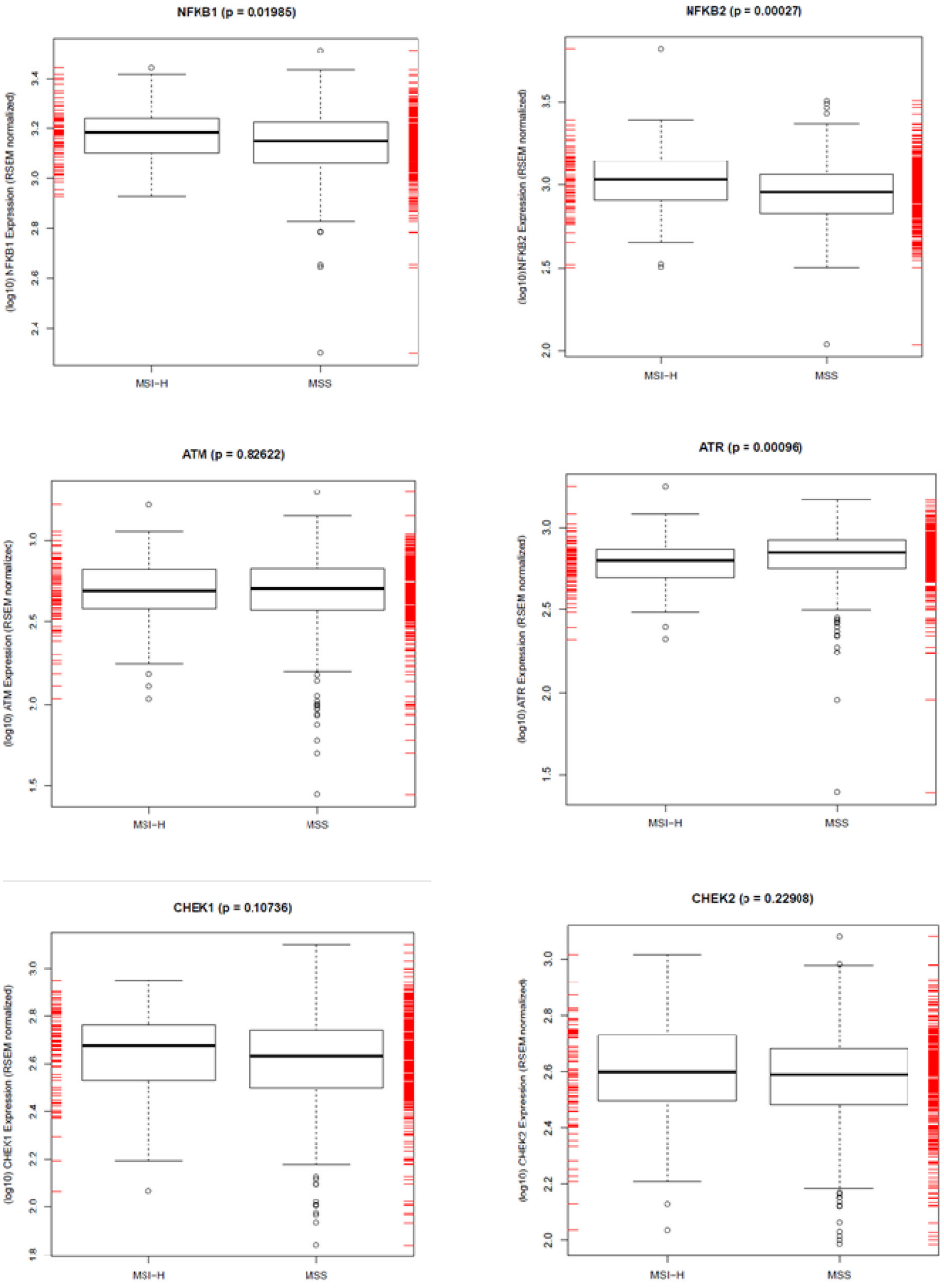
NFKB1 (p = 0.01985)
(log10) NFKB1 Expression (RSEM normalized)
MSI-H
MSS
NFKB2 (p = 0.00027)
(log10) NFKB2 Expression (RSEM normalized)
MSI-H
MSS
ATM (p = 0.82622)
(log10) ATM Expression (RSEM normalized)
MSI-H
MSS
ATR (p = 0.00096)
(log10) ATR Expression (RSEM normalized)
MSI-H
MSS
CHEK1 (p = 0.10736)
(log10) CHEK1 Expression (RSEM normalized)
MSI-H
MSS
CHEK2 (p = 0.22908)
(log10) CHEK2 Expression (RSEM normalized)
MSI-H
MSS

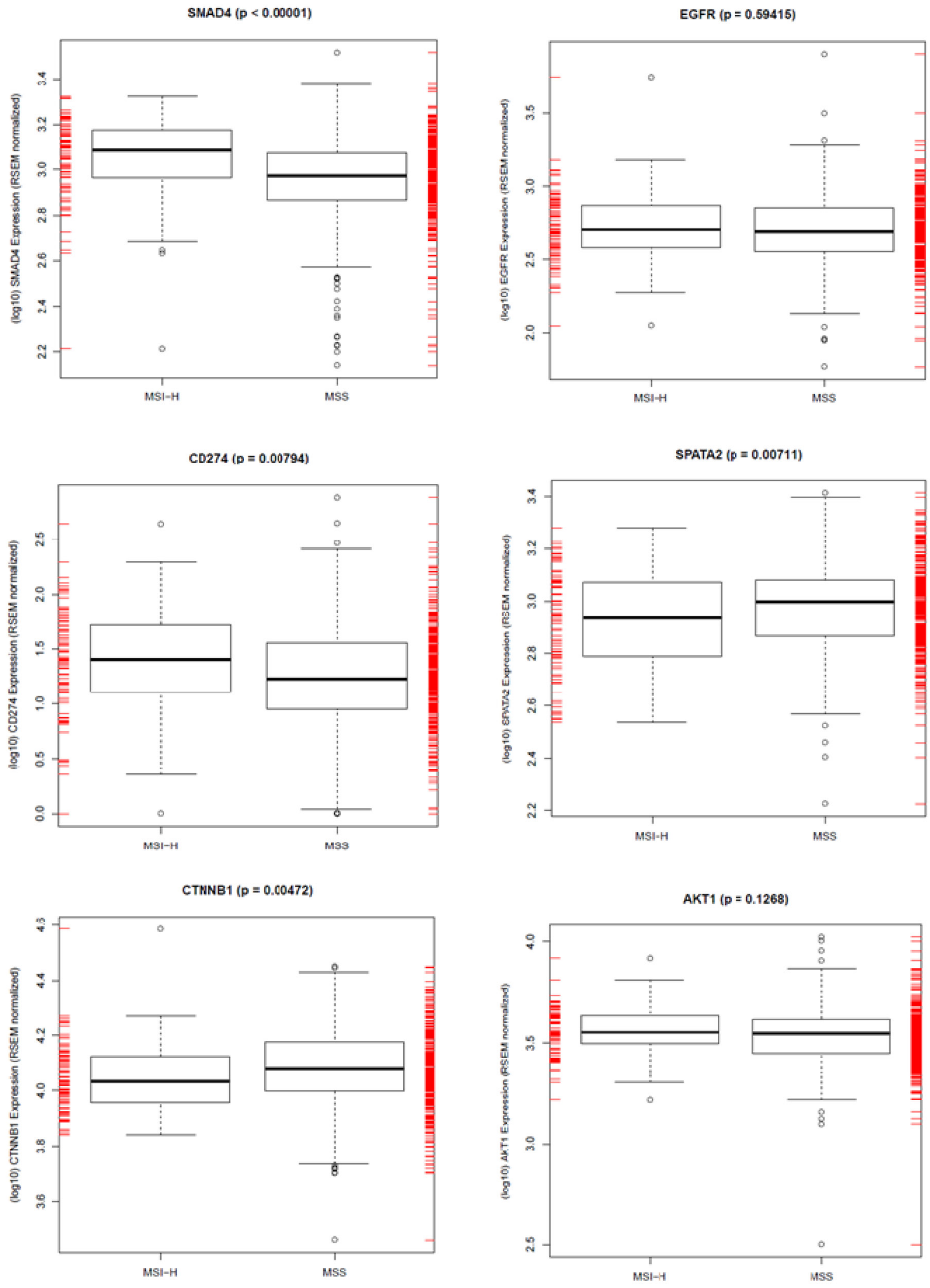
SMAD4 (p < 0.00001)
(log10) SMAD4 Expression (RSEM normalized)
2.2
2.4
2.6
2.8
3.0
3.2
3.4
MSI−H
MSS
EGFR (p = 0.59415)
(log10) EGFR Expression (RSEM normalized)
2.0
2.5
3.0
3.5
MSI−H
MSS
CD274 (p = 0.00794)
(log10) CD274 Expression (RSEM normalized)
0.0
0.5
1.0
1.5
2.0
2.5
MSI−H
MSS
SPATA2 (p = 0.00711)
(log10) SPATA2 Expression (RSEM normalized)
2.2
2.4
2.6
2.8
3.0
3.2
3.4
MSI−H
MSS
CTNNB1 (p = 0.00472)
(log10) CTNNB1 Expression (RSEM normalized)
3.6
3.8
4.0
4.2
4.4
MSI−H
MSS
AKT1 (p = 0.1268)
(log10) AKT1 Expression (RSEM normalized)
2.5
3.0
3.5
4.0
MSI−H
MSS

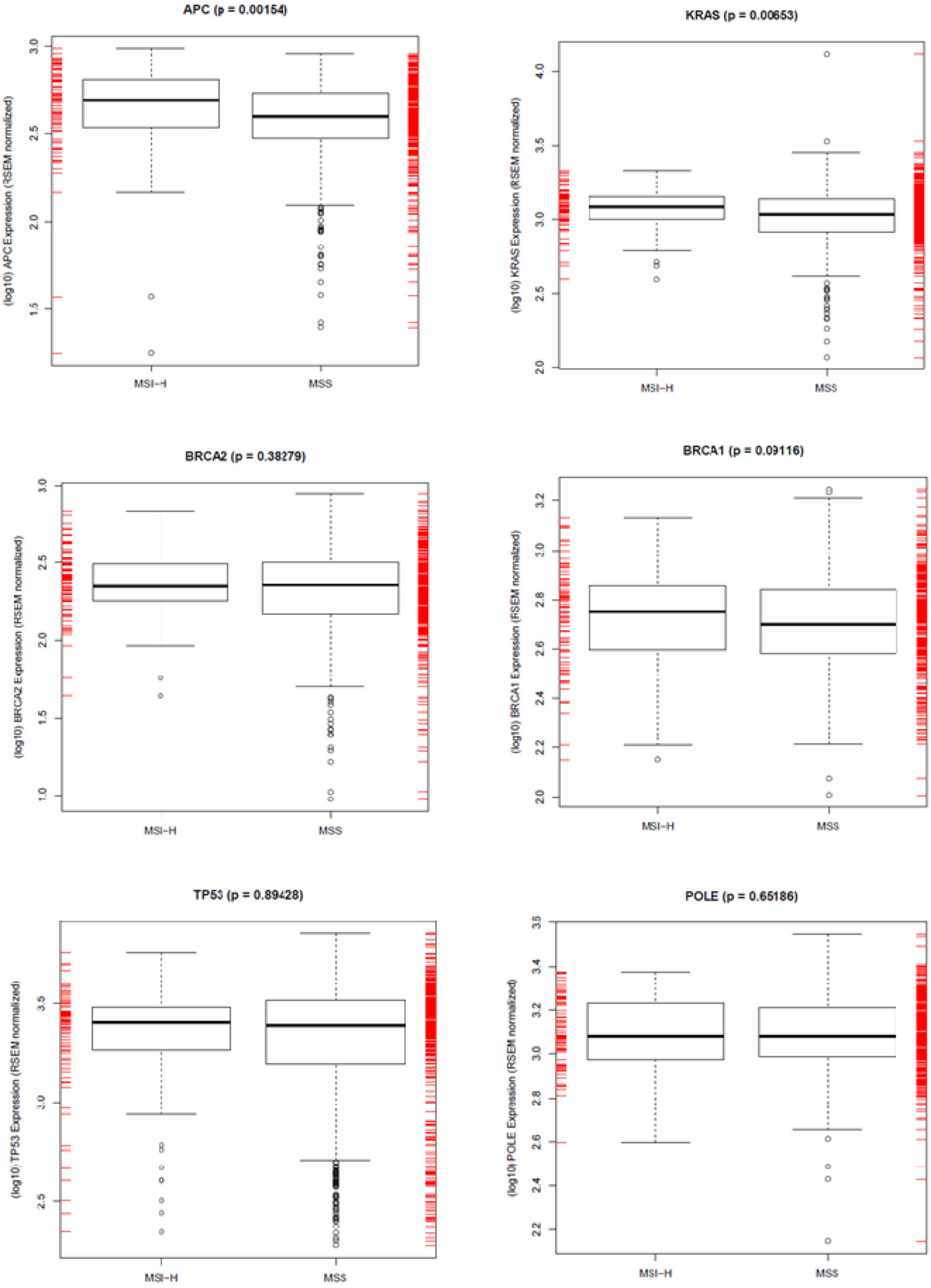


**Supplementary Figure S3.** Association between microsatellite instability and mRNA expression of NFKB1/2, ATM/ATR, CHEK1/2, SMAD4, EGFR, CD274, SPATA2, CTNNB1, AKT1, APC, KRAS, BRCA1/2, TP53, POLέ in CRC patients.

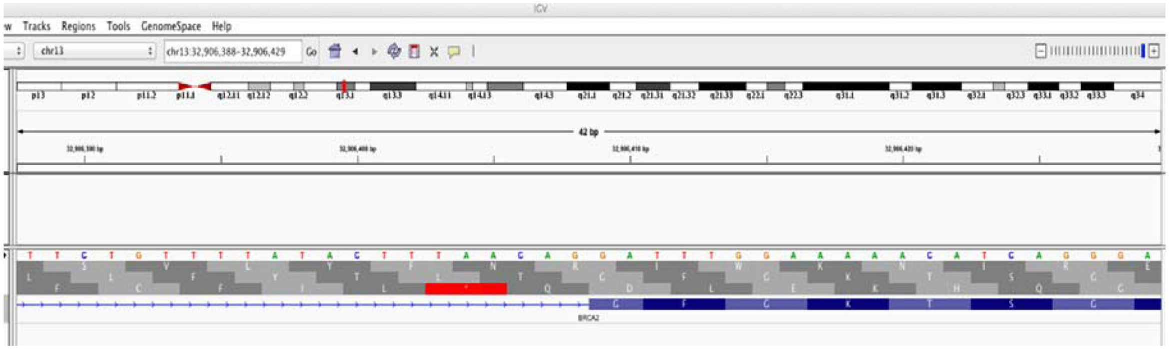


**Supplementary Figure S4.** One of the BRCA2 alterations was detected in splicing site. The red marked site is outside of the coding zone, considered the splicing site. Graphic representation of the specific site visualized with Integrative Genomics Viewer (IGV) software.

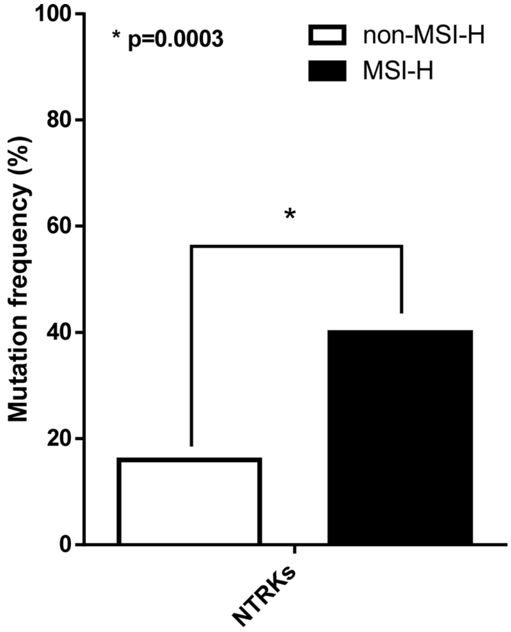


**Supplementary Figure S5.** High frequency of NTRK genes in MSI-H CRCS. Mutation frequencies in MSI-H and non-MSI-H groups are shown with p-values from Fisher's exact tests.

**For Supplementray Tables see in supplementary Files**
