# Supplementary material for: BRCA2, EGFR, and NTRK mutations in mismatch repair-deficient colorectal cancers with MSH2 or MLH1 mutations
Source: Oncotarget. 2017 May 23;8(25):39945–62. doi: 10.18632/oncotarget.18098 (PMC5522275; doi:10.18632/oncotarget.18098)
Supplement: Supplementary file 2 [file oncotarget-08-39945-s002.doc]

Supplementary Table S1. Polynucleotide repeats in coding BRCA2 are hotspots for alterations.

|  | **repeats** | **counts** | **Mutation involved** | **Involved Domain** |
| --- | --- | --- | --- | --- |
| **Mononucleotide** | AAAA  AAAAA  AAAAAA  AAAAAAA  AAAAAAAA  AAAAAAAAAA | 103  60  13  8  2  1 | K2017T, T1629A | Between BRC 7, 8  Interaction with NPM1    BRCA2DBD_OB3  BRCA2DBD_OB2 |
|  |  |  | Q321H, K965T, K722Q,K3360fs*23 |  |
|  |  |  | N319fs*5 , T3085A |  |
|  |  |  | T 3033fs*29 |  |
|  | TTTT  TTTTT  TTTTTT  TTTTTTTT  TTTTTTTTTT | 52  16  3  1  1 | I332M, H2415Y, H2415R, N337N, D1096N/E, F2349C, F2568C  Y232C | Interaction with FANCD2 |
|  | CCCC  CCCCC | 12  1 |  |  |
|  | GGGG | 5 |  |  |
| **Dinucleotide** | ACAC | 15  12 | R18C | Interaction with PALB2 |
|  | ACACA |  |  |  |
|  | CACA  CACAC  CACACA | 26  2  3 |  |  |
|  | TCTC  TCTCT  TCTCTC  TCTCTCT  TCTCTCTC | 19  10  4  1  1 | S3319Y  S3332Y  S1597Y  L2155H | Around NLS  Around NLS  Interaction with POLH |
|  | CTCT  CTCTC  CTCTCT | 23  1  2 | D878N | Interaction with NPM1 |
|  | AGAG  AGAGA  AGAGAG  AGAGAGA | 29  19  3  1 | E456D, E510* |  |
|  | GAGA  GAGAG  GAGGAG | 26  4  2 | E1879D | Between BRC 6, 7 |
|  | GTGT  GTGTG  GTGTGT | 13  5  1 |  |  |
|  | TGTG  TGTGT  TGTGTG | 25  10  1 |  |  |
|  | ATAT  ATATA  ATATAT | 42  11  4 | N1435H  H962H | Interaction with NPM1 |
|  | TATA  TATAT  TATATA | 22  7  1 | S1961G  Y2997* | BRCA2DBD_OB2 |
|  | GCGC | 1 |  |  |

| **Trinucleotide** | AACAAC  AACAACA  AACAACAA | 2  1  1 |  |  |
| --- | --- | --- | --- | --- |
|  | AAGAAG  AAGAAGA  AAGAAGAA | 3  4  3 | E1382K  E2599* | Interaction with POLH |
|  | AATAAT  AATAATA  AATAATAA | 7  2  1 | N987delN | Interaction with NPM1 |
|  | ACAACA  ACAACAA | 3  1 |  |  |
|  | ACGACG  ACTACT | 1  3 | R3384Q | NLS |
|  | AGAAGA  AGAAGAA | 9  4 | E2004E  E49* | Transcriptional activation |
|  | AGCAGC  AGCAGCA  AGCAGCAG  AGCAGCAGC | 1  3  1  1 |  |  |
|  | AGGAGG  AGGAGGA | 2  2 |  |  |
|  | AGTAGT | 1 |  |  |
|  | ATAATA | 1 |  |  |
|  | ATTATT  ATTATTA | 6  1 |  |  |
|  | CAACAA  CAACAAC | 5  1 |  |  |
|  | ATGATG  ATGATGA | 1  3 |  |  |
|  | ATCATC | 3 |  |  |
|  | CACCAC  CACCACC | 2  1 |  |  |
|  | CAGCAGCA | 1 |  |  |
|  | CATCAT  CATCATC  CATCATCA | 1  1  1 |  |  |
|  | CCACCA  CCACCACCAC | 2  1 |  |  |
|  | CGCG | 1 |  |  |
|  | CTACTA | 4 |  |  |
|  | CTCCTC | 1 | S3319Y | Around NLS |
|  | CTGCTG  CTGCTGC | 1  1 |  |  |
|  | CTTCTT  CTTCTTC | 1  2 |  |  |
|  | GAAGAA  GAAGAAG  GAAGAAGA  GAAGAAGAA | 15  1  1  1 |  |  |
|  | GATGAT  GATGATG  GATGATGA | 1  1  1 |  |  |
|  | GCAGCA  GCAGCAG | 1  1 |  |  |
|  | GGAGGA  GGAGGAG | 2  1 |  |  |
|  | GGTGGT | 2 |  |  |
|  | GTAGTA  GTAGTAG | 1  1 |  |  |
|  | GTCGTC | 1 |  |  |
|  | GTGGTG | 2 |  |  |
|  | GTTGTT | 2 |  |  |
|  | TAATAA  TAATAAT  TAATAATA | 2  1  1 | F701C | Interaction with NPM1 |
|  | TACTAC  TACTACT | 2  1 |  |  |
|  | TAGTAG  TAGTAGT | 1  1 |  |  |
|  | TATTAT  TATTATT | 1  1 |  |  |
|  | TCATCA | 1 |  |  |
|  | TCCTCC | 1 |  |  |
|  | TCTTCT | 1 |  |  |
|  | TGATGA  TGATGAT | 5  1 |  |  |
|  | TGCTGC | 1 |  |  |
|  | TGGTGG | 2 |  |  |
|  | TGTTGT  TGTTGTT | 1  2 |  |  |
|  | TTATTA | 3 | F701C | Interaction with NPM1 |
|  | TTCTTC  TTCTTCT | 7  1 |  |  |
|  | TTGTTG | 2 |  |  |

*Red-colored mutations are predicted to be damaging.

** The underlined mutations exist within the underlined domains.
